# Acceptability of neonatal circumcision by pregnant women in KwaZulu-Natal, South Africa

**DOI:** 10.4102/curationis.v38i1.1433

**Published:** 2015-03-30

**Authors:** Rogerio Phili, Quarraisha A. Karim

**Affiliations:** 1 Department of Public Health Medicine, School of Nursing and Public Health, University of KwaZulu-Natal, South Africa; 2 Centre for the AIDS Programme of Research in South Africa, University of KwaZulu-Natal, South Africa

## Abstract

**Background:**

Studies on voluntary medical male circumcision (VMMC) have provided convincing evidence on its efficacy to provide partial protection against female-to-male HIV transmission in circumcised men. The World Health Organization and UNAIDS subsequently formulated recommendations for VMMC implementation that included implementation of neonatal medical male circumcision (NMMC) to all infants up to two months old. Knowledge regarding the acceptability of NMMC by pregnant women who are candidates for granting of consents for NMMC procedures or its ideal placement within health programmes is low.

**Objectives:**

We sought to establish NMMC acceptability by pregnant women and the feasibility of its integration within Maternal, Child and Women's Health (MCWH) programmes to inform implementation guidelines.

**Method:**

Nurses and counsellors at two public health facilities were trained to provide NMMC counselling and offer NMMC to 1778 pregnant women presenting for antenatal care services. Univariate and bivariate analyses were performed on data collected on NMMC acceptance and refusals. Thematic analysis was also performed on qualitative reasons for refusals.

**Results:**

Acceptability of NMMC by women was high (82.9%). Refusals resulted from the need for consultations with partners and/or family members prior to consenting (41.3%), fear of the procedure (23.8%), cultural reasons (15.9%) and no reasons given (15.3%).

**Conclusion:**

The acceptability of NMMC by pregnant women and its integration with MCWH services was feasible. However socio-cultural factors, including the need for further consultation prior to consenting for NMMC procedures and preference of traditional circumcision by some women, need to be addressed in order to increase uptakes.

## Introduction

Voluntary medical male circumcision (VMMC) has been shown to reduce the risk of female-to-male sexual transmission of HIV by approximately 60% in three randomised controlled trials (RCTs) conducted in sub-Saharan Africa (Auvert et al. 2005; Bailey et al. 2007; Gray et al. 2007). Following the results of the RCTs, as well as the World Health Organization (WHO)/Joint United Nations Programme on HIV/AIDS (UNAIDS) recommendations (WHO & UNAIDS 2011), many countries embarked on the implementation of VMMC as part of their HIV prevention strategies. In order to achieve optimum medium- to long-term public health benefits of VMMC, the WHO and UNAIDS recommended the concurrent implementation of neonatal medical male circumcision (NMMC) of infants up to two months old alongside adult VMMC in settings of high HIV prevalence and low circumcision prevalence. NMMC is also considered to be safer, more cost-effective when compared to adult VMMC (Binagwaho et al. 2009) and eliminates risk compensation (an increased sexual risk-taking because of perceived lower risk of infection) (Cassel et al. 2006).

Despite the public health advantages, the implementation of NMMC for HIV infection mitigation faces some ethical and human right arguments (American Academy of Pediatrics 2005; Lerman & Liao 2001). Most ethical issues are related to the validity of the parental consent for the procedure based on the infant's inability to give its own consent (Hellsten 2004). Others argue that the existing scientific evidence is insufficient to recommend routine NMMC for HIV prevention later in life.

The legal opposition is based mainly on *The Children's Act* No. 38 of 2005 (Sloth-Nielsen 2010), which is interpreted as indicating that circumcision of male children under the age of 16 years is prohibited unless it is done for medical or religious reasons. However, in routine health practice, parents regularly practise proxy decision making for their children when choosing other (and sometimes invasive) therapeutic interventions, as well as some mandatory procedures, such as vaccination of children.

In most child health services, mothers in particular are the main decision makers regarding health services for their children. Other studies indicate the presence of social, economic, gender and age factors that influence women's decision making in HIV prevention programmes (Maputle & Jali 2008; Thairu et al. 2005). Much has been published regarding the acceptability of circumcision in the general population (Gray, Wawer & Kigozi 2013; Mavhu et al. 2012; Westercamp & Bailey 2007), but not much is known about NMMC acceptance, particularly by expectant women in non-circumcising communities who are candidates for NMMC consent-granting decisions. The ability of women to grant NMMC consent is critical in ensuring the uptake of this intervention and in determining the feasibility of its integration with other Maternal, Child and Women's Health (MCWH) programmes that they and their children routinely attend.

We aimed to explore the acceptability of NMMC amongst pregnant women attending select antenatal care (ANC) clinics at communities with low circumcision prevalence. The study was conducted as a subcomponent of a larger project to determine the feasibility of implementation of a VMMC programme at public health facilities in KwaZulu-Natal (KZN).

## Research design

### Research approach and method

This was a short operational research (OR) study of a prospective longitudinal nature and employing quantitative and qualitative methods.

#### Population and sampling

Two health facilities were used: a primary health centre in the Midlands region of KZN (Midlands Community Health Centre [MCHC]) in Umgungundlovu health district and a semi-private district hospital in Durban (Durban District Hospital [DDH]) in Ethekwini health district. The MCHC was based in a densely-populated urban setting but serviced a mix of urban and rural patients. Most patients who use the facility are predominantly indigent people residing in townships and scattered rural settlements around Pietermaritzburg. The DDH facility is a 24-hour institution providing a comprehensive range of primary and district health services to mainly peri-urban and urban residents in the outer west area of the city. Its patients are mainly the working class who reside in densely-populated townships and informal settlements.

Between November 2010 and May 2011, pregnant women who attended ANC services at the study facilities were approached and asked to participate in the study. Women who agreed to participate were asked to sign an informed consent for participation by the counsellors.

#### Procedures

We began by amending ANC patient information collection tools (registers) to include sections on the offering and acceptance of NMMC. Three columns were added to the register in order to record the responses of the prospective mothers to two closed-ended questions on whether they accepted or refused to have their male newborn babies circumcised and the reasons for refusal thereof (if applicable). The reasons for refusal were pre-determined and coded to represent reasons such as fear of the procedure, cultural reasons, further consultation with partner of family, no reason indicated and other reasons to be specified.

The health providers (nurses and counsellors) were then trained on all aspects of NMMC and its desired use for HIV prevention later in life. The health providers were instructed to offer NMMC counselling and to ask the pregnant women presenting at the clinics whether they were willing to accept NMMC (willing to grant informed consent) for their newborn males in addition to the HIV counselling and testing services routinely offered to these clients. The acceptances and refusals of the procedure were recorded in the registers, along with the reasons for refusals. Women were encouraged to elaborate on the reasons given if they so wished. The health providers were issued with notebooks to record any additional information provided by the pregnant women, including the explanations given for ‘other’ reasons.

#### Data treatment

The data collected from the registers were entered onto an SPSS database version 21 (IBM Corp., Armonk, NY 2012). Univariate and bivariate analysis was undertaken using Pearson's χ^2^. A *p*-value of < 0.05 was considered statistically significant. The primary outcome of the study was the acceptance rate and the reasons for refusal of NMMC. Thematic analysis was conducted on the qualitative data on the ‘other’ reasons for refusal and the elaborate explanations given by some women in accordance with the aim of the study.

## Results

A total of 1778 women were registered between the two facilities. Data on the initial visit were considered for women who were repeat attendees. Most participants (61.4%, *n* = 1091) were from the MCHC and 38.6% (*n* = 687) were from the DDH. The HIV seroprevalence amongst the women was 27.6% (33.2%, *n* = 357 for MCHC and 18.4%, *n* = 119 for DDH). The median age of the participants was 25 years (range: 12–47).

The overall acceptance rate of NMMC amongst women was 82.9% (*n* = 1073: MCHC = 59.7%, *n* = 641; DDH = 40.3%, *n* = 432). The NMMC acceptance within study site was also high (MCHC = 82.2%, *n* = 641; DDH = 84.1%, *n* = 432) There was no statistically significant association between the acceptance of NMMC by women and the site of presentation (*p* = 0.382). Similarly, no association was found between the NMMC refusals and the site (*p* = 0.388). Most refusals were because of: the women being required to consult with partners and/or family before they could consent to the procedure (41.3%; *n* = 78); fear of the procedure (23.8%; *n* = 45); cultural reasons (15.9%; *n* = 30); and no reasons given (15.3%; *n* = 29).

An independent samples *t*-test revealed that there was no statistically significant difference in terms of age between the women who accept NMMC (*p* = 0.089) and those who declined (*p* = 0.083). The mean age of the women who were able to consent to NMMC was 26 years, whilst it was 25 years for those who declined.

Twenty-six records of the other NMMC refusal reasons given were analysed and two distinct themes of influences on NMMC acceptance emerged: (1) socio-cultural factors influencing NMMC acceptance; and (2) conceptual knowledge factors leading to reservations about accepting NMMC procedures. Socio-cultural factors were pronounced by statements such as:

‘My husband will never agree to that; where will the tissue [*foreskin*] go? This is a big issue as a ceremony needs to be done and the tissue buried in the yard.’ (26-year-old female, MCHC)

Some of the cultural beliefs and service mistrust issues were strong, despite the knowledge given by health providers. This was evidenced by the perceptions that traditional circumcision (TC) has the same benefit as NMMC and the stigma attached to circumcision at health institutions:

‘No, my son will go to the mountain as per standard practice when he is older.’ (29-year-old female, DDH)

‘I heard that they use a clamp to circumcise boys and that it's painful... I don’t want my child to go through that.’ (18-year-old ANC client, female, DDH)

## Ethical considerations

Ethical clearance to conduct this study was obtained from the University of KwaZulu-Natal Biomedical Research Ethics Committee (Reference No: BF035/010) and the Department of Health KwaZulu-Natal. Approval was also granted by the health institutions where the study was conducted.

## Trustworthiness

The honesty of information given by participants was ensured initially by voluntary acceptance to participate or refuse participation in the study, thereby ensuring that only those who were genuinely willing to take part and prepared to give data freely were enrolled. The reliability and credibility of the data were ensured through the use of the following initiatives:

- Use of recognised, appropriate data collection tools and methods.
- Offering of all women presenting for ANC services participation and therefore eliminating researcher bias in selection of participants.
- Triangulation via use of different counsellors to ensure verification of individual viewpoints.
- Iterative questioning during data collection, where responses previously given participants were revisited during the follow-up interviews and through rephrasing questions to uncover inconsistent information.
- The study results were subsequently compared with those of other studies to establish congruency of findings.
- The counsellors who conducted data collection and interviews were experienced in their fields, therefore ensuring quality of data collection.

The information given to participants on NMMC is from standard WHO protocols and is the same for all health institutions throughout the world. Therefore the findings may be transferable to other similar situations or to facilities with characteristics similar to those described in this study.

## Discussion

Despite the current debates on the ethics of the parental consent to the NMMC procedure, there remains a high acceptability of NMMC by pregnant women who are candidates for granting informed consent for NMMC in the two non-circumcising communities studied. The high acceptability of the intervention by women makes its integration with MCWH programmes both logical and feasible, as it would allow for active recruitment of infants for NMMC during the normal course of attending ANC services. The post-surgical follow-up visits would also be easier as women routinely bring their children post-delivery for ongoing child health services. The high acceptability of NMMC by women is consistent with findings in other studies (Mavhu et al. 2012; Westercamp & Bailey 2007), although most were conducted amongst women in general populations.

### Reasons for non-acceptance

As observed in other studies, women were willing to grant consent for their children because of the perceived health benefits of NMMC (Ahaghotu et al. 2009). Contrary to findings in other studies that mainly indicate cultural, economic and knowledge factors as the main inhibitors to women's capacity to grant consent (Mavhu et al. 2012; Thairu et al. 2005; Rediger & Muller 2013), we found that the need for further consultation was the main reason for NMMC refusal or deferment of consent by the women in these communities. Therefore, counselling services for women need to incorporate men as partners in order to ensure good uptake of NMMC procedures. Other studies also emphasise the importance of effective counselling as a pre-requisite to higher levels of acceptance (Brown & Brown 1987).

The delayed decision making because of the need for further consultation has been shown to be positively associated with a woman's age, education and employment status (Acharya et al. 2010; Duong, Lee & Binns 2005; Thairu et al. 2005;). Duong et al. (2005) found that pressure from family members negatively influenced decisions on infant-feeding practices, particularly for young mothers. Other studies indicate that painless circumcision is possible in almost all newborns if it is performed during the first week after birth (Banieghbal 2009). Any delays in consenting to NMMC may lead to the situation where circumcision procedures have to be deferred for later in the child's life. This would necessitate the use of anaesthetics and result in other cost implications of the procedure that would have been avoided by timely consent granting practices.

We found that the age of the woman is not a factor in the decision-making process for granting of consent in this study. Another factor that needs to be addressed through counselling is the fear of the procedure that was expressed by the women. This factor was mainly influenced by the stories that some women had heard regarding the complications caused by the use of the Tara Klamp^® ^device to circumcise adolescents at some health facilities.

### Socio-cultural and knowledge factors influencing acceptability

The study reveals the presence of a degree of confusion regarding the role played by TC and the objectives of the NMMC strategy. There were perceptions that NMMC would be done by individuals who wanted to avoid TC rather than because of the medical benefits of NMMC. This was viewed as ‘weakness’ by the proponents of TC who also believed that the passage to manhood inferred by TC should involve the ability to withstand the pain of the procedure. The ability to withstand pain is a criterion for successful completion of TC in some cultures (Westercamp & Bailey 2006).

The unacceptability of NMMC by some women, based on the belief that the circumcision procedure should be done at the adolescent stage, is another evidence of the perception held by some women that the TC was similar to VMMC, and that NMMC was being introduced as an easy option for TC. Some women had strong beliefs that traditional rites had to be observed after any circumcision procedure and that this would be missing in the NMMC procedures. Other authors also note that the use of such reasons is predominant in settings where circumcision of young men is central to the concepts of masculinity and maturity (Kalichman 2010; Marck 1997). The need for cultural rites was also shown by the insistence that traditional ceremonies, such as the burial of the foreskin in the yard, need to be performed after circumcision. It is important to provide comprehensive counselling on the differences between the two practices in order to avoid drawing of parallels between them. The counselling of women needs to be structured such that attention is paid to cultural constructs and the strong attachments to the traditional procedure displayed by some women.

Other knowledge gaps identified included the lack of skills to communicate the decision to circumcise with other family members and with the children when they grew older. Some parents and guardians may be dissuaded from having their sons circumcised, as consent processes may involve conveying information about sexuality and HIV and AIDS to children (Rennie, Muula & Westreich 2007).

### Ethical issues

There were no ethical issues relating to NMMC consent that were revealed by this study. In addition, the willingness of health workers to offer NMMC to clients did not appear to be influenced by ethical reservations, as was also noted by Lannon et al. (2000). The debate on the ethical considerations currently appears to be at administrative and policy levels only.

### Feasibility of integration with Maternal, Child and Women**’**s Health programmes

The implementation of NMMC for HIV prevention is currently provided on a limited basis in KZN and is mainly confined to medical indications. The non-implementation of this intervention appears to be the trend in most countries, mainly because of the non-review of policies to integrate NMMC with existing health services and the cultural and health systems-related issues (Kebaabetswe et al. 2003; UNAIDS 2012).

The study shows that it would be easier to integrate NMMC with the MCWH programmes as NMMC counselling could be offered as a package of services for women attending ANC and child health services. Furthermore, the data collection tools can be integrated easily and it may be also easier for prospective parents to liaise with single health providers rather than having to attend a different service to access NMMC.

## Limitations of the study

The study comprised predominantly black African females because of the socio-demographics of the communities wherein the study facilities are based. The communities studied were historically non-circumcising; therefore findings may be different in settings where circumcision is practised routinely and is a rite of passage of young males to manhood. The counsellors’ time was also limited because of the high volumes of clients presenting for services, which may have limited the amount of qualitative information sought from participants who refused NMMC. The study was also conducted at two health facilities in a predominantly urban/peri-urban settings.

## Recommendations

There is a need for immediate integration of NMMC counselling with HIV pre-test counselling and other MCWH services at public health facilities. However, the NMMC counselling process should involve men as partners and other relevant family members in order to expedite timeous NMMC consent decision making. This will prevent unnecessary delays in the scaling-up of NMMC and prevent undue deferment of circumcision procedures. Cultural issues and differences between NMMC and the TC issues need to be included and clarified in the counselling sessions in order to allow clients to make informed decisions about NMMC.

## Conclusion

The study shows a high acceptability of NMMC by pregnant women, indicating the probability of a high uptake of the intervention at the public health facilities in the province. Furthermore, the integration of NMMC strategy with the existing MCWH services is feasible. However, some women may be reluctant to consent to NMMC procedure because of the need for further consultation with family members and the perceived cultural change associated with circumcising infants at medical facilities. To make an informed choice, parents of all male infants should be given accurate and unbiased information and be provided the opportunity to discuss this decision. The integration of NMMC with MCWH services could also facilitate the recruitment of children in school who are often missed by HIV prevention programmes as they present for services such as immunisations.
